# Supplementary material for: Endogenous stimulus-powered antibiotic release from nanoreactors for a combination therapy of bacterial infections
Source: Nat Commun. 2019 Oct 2;10:4464. doi: 10.1038/s41467-019-12233-2 (PMC6775118; doi:10.1038/s41467-019-12233-2)
Supplement: Supplementary file 1 — Supplementary Information [file 41467_2019_12233_MOESM1_ESM.pdf]

SUPPLEMENTARY INFORMATION

Endogenous stimulus-powered antibiotic release from nanoreactors for a combination therapy of bacterial infections

Yang Wu , Zhiyong Song, Huajuan Wang, and Heyou Han

Materials and Methods.

Materials Lauric acid (LA), stearic acid (SA), lecithin (Lec)，rifampicin (RFP), DSPE-PEG 3400 and 8-aminonaphthalene-1,3,6-trisulfonic acid disodium salt (ANTS) were purchased from Aladdin Industrial Corporation (Shanghai, China). Calcium hydroxide [$Ca(OH)_2$], 30% aqueous hydrogen peroxide(H_2O_2) and polyvinyl alcohol (PVA) were purchased from Sinopharm Chemical Reagent Co., Ltd. p-xylene-bis-pyridinium bromide (DPX) was purchased from Tokyo chemical industry Co., Ltd. Propidium iodide (PI), 4'-6-diamidino-2-phenylindole (DAPI) products and Hydrogen Peroxide Assay Kit were purchased from Beyotime Institute of Biotechnology (Shanghai, China). Hla, Anti -Staphylococcalα-toxin (α-Hemolysin) antibody produced in rabbit and HRP-conjugated Goat Anti-Mouse lgG were purchased from Sigma-Aldrich. Goat Anti-Rabbit lgG H&L (40nm Gold) was purchased from abcam. Gram-positive *B. subtilis* (AB 90008) was acquired from China Center for Type Culture Collection. MRSA was isolated from clinical. 4% paraformaldehyde PBS buffer was purchased from Wuhan Google Biotechnology Co., Ltd. H&E and Masson staining were performed by Wuhan Google Biotechnology Co., Ltd. The BABL/c mice were purchased from the Animal Experimental Center of Huazhong Agricultural University.

Synthesis and characterization of the CaO_2 nanoparticles. Briefly, 6 g $Ca(OH)_2$ was dissolved in 120 mL of DI water and mixed well, followed by the addition of 0.096 g PVA to the suspension and incubation for 50 minutes at room temperature. Subsequently, the 63.46 mL 30% aqueous H_2O_2 solution was slowly added dropwise

into the mixed solution and well mixed under constant stirring on ice bath for 15 minutes until the complete reaction of calcium salt and H_2O_2 to form stable nano-scale CaO_2 (calcium peroxide) particles. After the reaction, the mixture was centrifuged, and the product was washed with DI water and isopropyl alcohol. Finally, the product was vacuum-filtered and dried to obtain a nano-sized CaO_2 powder[1].

The result was shown in Supplementary Fig. 16a. The TEM (JEM-2010) image showed that the size of CaO_2 was approximately 100 nm. In Supplementary Fig. 16b, XRD analysis was done to identify the material of CaO_2 powders. The three dominant peaks: 2θ= 30.3, 35.4, 47.0 well matched the XRD of CaO_2 (card number 03-0865)[2]. The XRD result strongly proved the formation of the CaO_2 nanoparticle compound by this procedure.

Toxin Loading Analysis. BCA Protein Assay Kit was used to quantitative detection of the adsorption of toxins by materials. 200μL different concentrations (500, 200, 100, 50, 25μg/mL) of RFP-CaO_2@PCM@Lec added with 10μL 400ug/mL toxin interact at 37 °C for two hours, as PBS for control. Then wash off unadsorbed toxins with PBS and detection of toxin concentration at 462 nm using the BCA protein assay kit.

An immunogold staining assay was carried out to confirm insertion of toxin onto the RFP-CaO_2@PCM@Lec nanoparticles[3]. 200 μL 500 μg/mL RFP-CaO_2@PCM@Lec added with 10μL 400ug/mL toxin interact at 37 ℃ for two hours, as no toxin for control. The solution were then washed before subjecting to blocking with 1wt% bovine serum albumin (BSA), primary immunostaining with polyclonal rabbit

anti-Staphylococcal α-toxin (α-Hemolysin) antibody (Sigma-Aldrich, MFCD00162866, 1:10000) for 30min and secondary staining with gold Anti-Rabbit IgG H&L (40 nm Gold) preadsorbed (abcam, ab119180, 1:100) for 30min.The solution was dropped onto the glow-discharged carbon-coated 400-mesh copper grid (Electron Microscopy Sciences).

RFP release assay. The RFP-CaO_2@PCM@Lec and the RFP@PCM@Lec were evenly dispersed in the DI water, with the toxin added or not, and the groups consisted of:1) RFP-CaO_2@PCM@Lec+toxin, 2) RFP-CaO_2@PCM@Lec+DI; 3) RFP@PCM@Lec+toxin; 4) RFP@PCM@Lec+DI. The samples were centrifuged to collect the supernatant for UV−vis absorption spectroscopy analysis (Thermo Nicolet, United States) at different time points (30, 60, 90, 120, 150 min).

H_2O_2 production assay. The RFP-CaO_2@PCM@Lec was evenly dispersed in the DI water, then the toxin was added or not. Next, the samples were centrifuged to collect the supernatant for detection by using the Hydrogen Peroxide Assay Kit at a different time point (30, 60, 90, 120, 150, 180, 360, 540, 720 min) according to the manufacturer's instructions.

Bacterial culture. The antimicrobial activity of the different materials was evaluated by the plate dilution method as follows. The response of PCM nanoparticles to the toxin was explored under four different experimental groups: Ⅰ RFP-CaO_2@PCM@Lec nanoreactors+MRSA, Ⅱ PCM@Lec+MRSA. Ⅲ RFP-CaO_2@PCM@Lec nanoreactors +*B.subtilis*, Ⅳ RFP-CaO_2@PCM@Lec nanoreactors + *B. subtilis*+ toxin; were treated separately with MRSA and *B. subtilis*

at different conditions and concentrations (100, 50, 25, 12.5, 6.25 μg/mL). The MRSA and *B. subtilis* were collected by centrifugation at 3000 rpm for 3 min, then the bacteria were resuspended in water and adjusted to 10^8 CFU/mL. Next, 200 μL of 10^8 CFU/mL bacteria was incubated with different concentrations of RFP-CaO_2@PCM@Lec nanoreactors and PCM@Lec, followed by the addition of toxin or not, at 37°C for 2 h at 120 rpm. To evaluate the bacterial mortality, the treated bacteria were diluted and uniformly plated in Luria-Bertani (LB) solid medium to grow for 24 h at 37 °C. Meanwhile, colony forming unit (CFU) was counted and compared with the control plate. Each treatment was prepared in triplicate and the mean values were compared with each other.

Live/Dead staining assay. In order to gain a more intuitive performance of the bactericidal effect, live/dead staining assay was performed. The bacterial cells were stained with PI (100 μg/mL) and DAPI for 15 and 5 min in the dark, respectively. Fluorescence images were taken on an Olympus BX40 fluorescence microscope during a single batch experiment at 400× magnification (Nikon, Japan).

***In vitro* antibacterial experiments.** To explore the antibacterial effect of different PCM nanoparticle materials against MRSA, 200 μL of 10^8 CFU/mL MRSA was incubated separately with 200 μL of 100 μg/mL of RFP-CaO_2@PCM@Lec nanoreactors, RFP@PCM@Lec, CaO_2@PCM@Lec and PCM@Lec under 37°C for 2 h at 120 rpm. The optical density (OD600) of the treated MRSA was measured at the indicated time interval during the logarithmic phase.

In Vitro safety. Assess the toxicity of RFP-CaO_2@PCM@Lec nanoreactors, the Vero cells were plated into 96-well plates and incubated with different concentration (500, 200, 100, 50, 25) of RFP-CaO_2@PCM@Lec nanoreactors, After 24 h of incubation with the different samples. Cell viability was assayed using an MTT reagent. Untreated cells were used as the 100% viability control.

In Vivo safety. The BALB/c mice (6-8 weeks old) were first shaved to remove the hair on the back. Subsequently, 200μL 100μg/mL of RFP-CaO_2@PCM@Lec nanoreactors (20 μg) was injected subcutaneously, as the PBS for control. After 24h, the mice were euthanized, and the internal organs (heart, liver, spleen, lung, kidney) were collected for histological processing by hematoxylin and eosin (H&E). The plasma was collected for biochemical indicator detection (ALB, ALP, ALT, AST, A/G, BUN, GLOB, TP). Assess the toxicity of RFP-CaO_2@PCM@Lec nanoreactors (100 μg), toxin(4 μg), heated toxin (4 μg ,70℃ inactivated for 1h) and nano-toxin (4μg toxin absorbed by 100μg RFP-CaO_2@PCM@Lec), the PBS as control. BALB/c mice were first shaved to remove the hair on their back. These materials were injected subcutaneously. After 24h, the mice were euthanized, and skin samples at the site of injection were collected for histological processing by hematoxylin and eosin (H&E) and TUNEL.

Histology analysis. The mice were put to death and the wound tissues were harvested at 4 and 10 days post infection. The wound tissues treated with different nanoparticles were fixed in 4% paraformaldehyde PBS buffer and stained with H&E and Masson. The samples were examined in Wuhan Google Biotechnology Co., Ltd.

Disposal of the experimental mice. In order to reduce the impact of experimental mice on the environment, the wound tissues were harvested and the experimental mice were collected and sterilized.

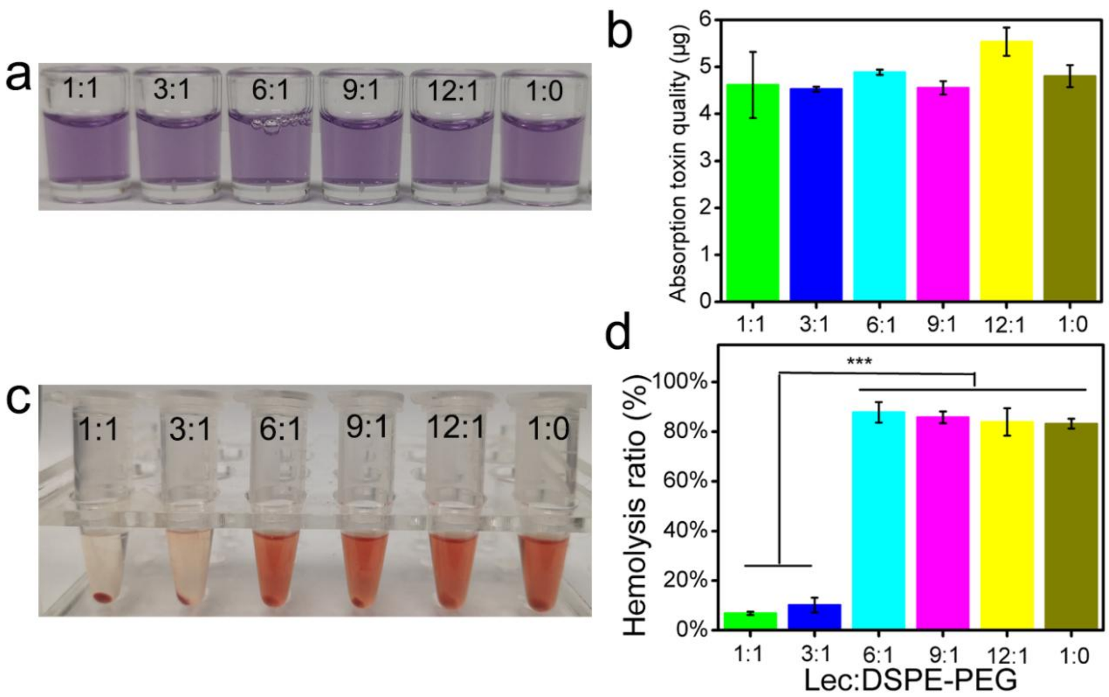

Supplementary Fig. 1 The different mass ratios for lecithin to DSPE-PEG. The toxin absorption efficiency (a and b) and the hemolysis ratio (c and d) of different ratio nanoformulations. Error bars = standard deviation (n=3). The mean value was calculated by the t test (mean ± SD). *p < 0.05, **p < 0.01, ***p < 0.001, compared with the indicated group. Source data are provided as a Source Data file.

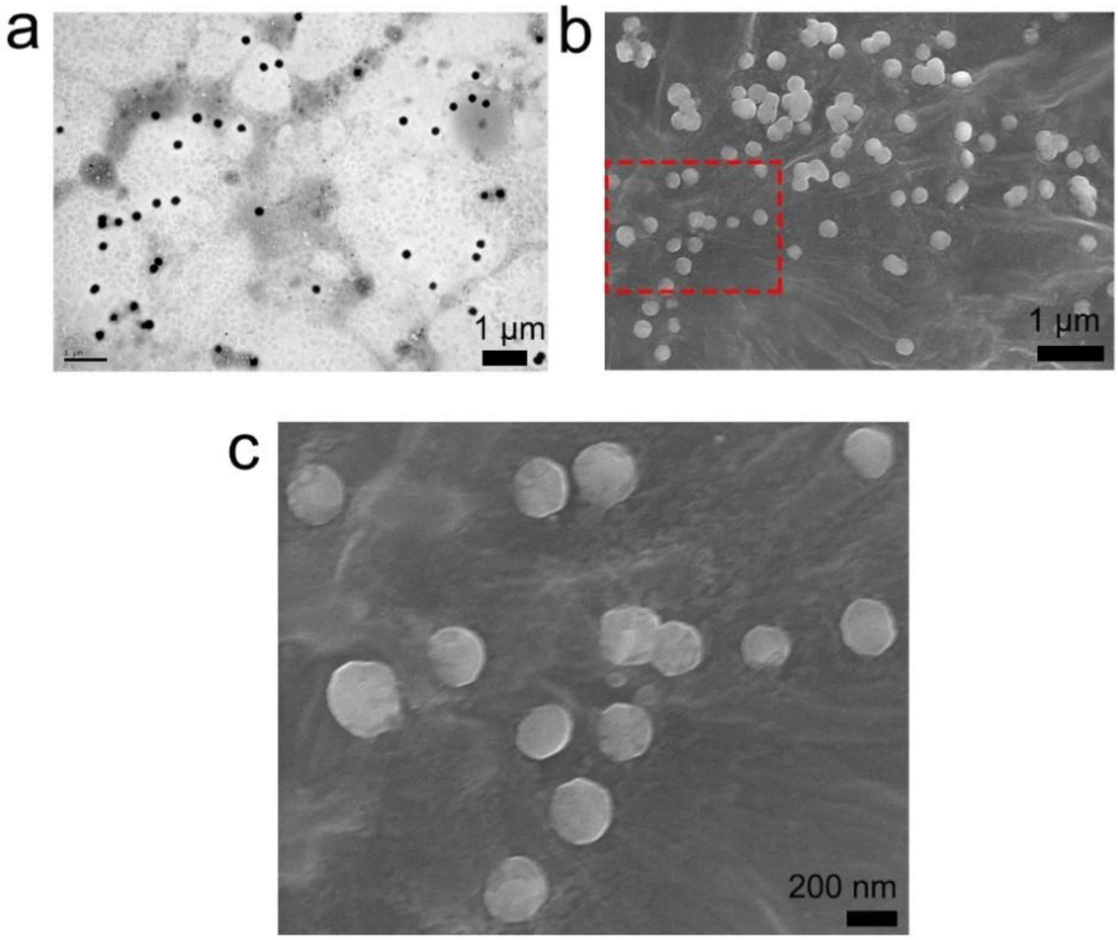

Supplementary Fig. 2. (a) TEM and (b, c) SEM image of the nanoreactors. Source data are provided as a Source Data file.

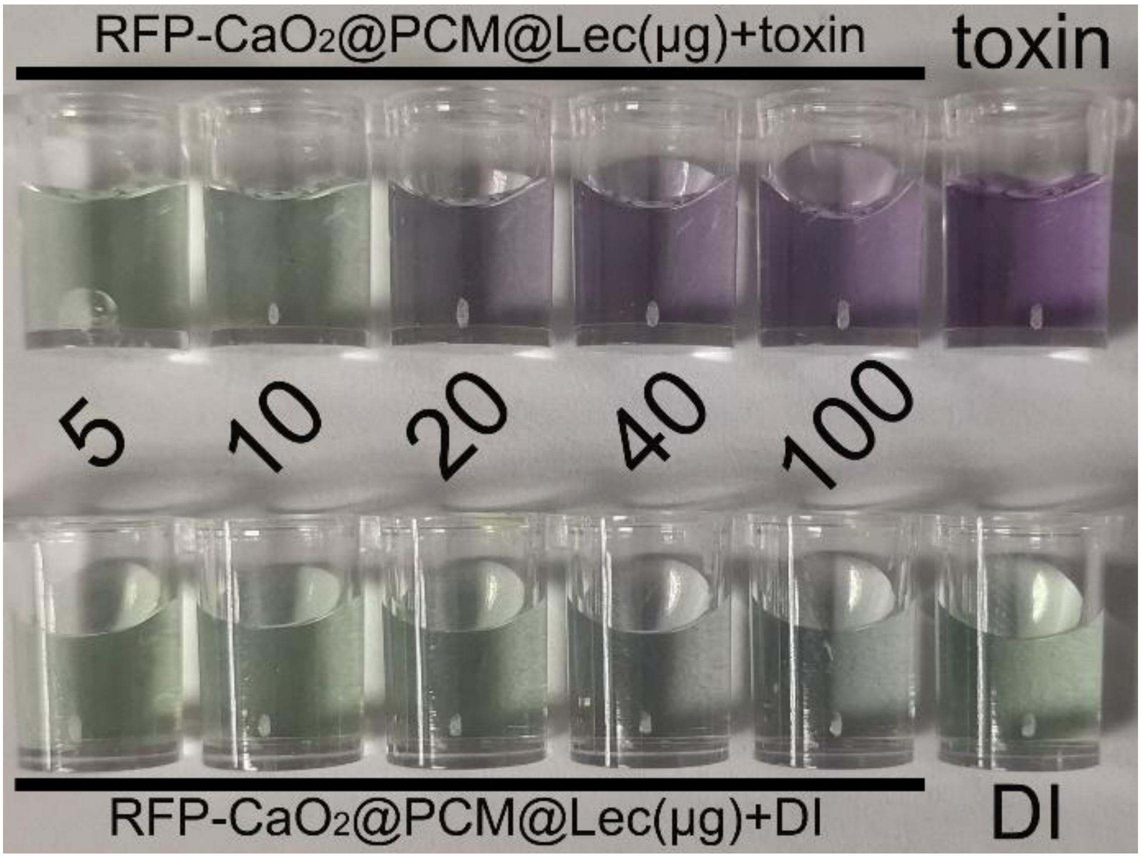

Supplementary Fig. 3. Detection of the adsorption of toxins by different quality materials using BCA Protein Assay Kit. Source data are provided as a Source Data file.

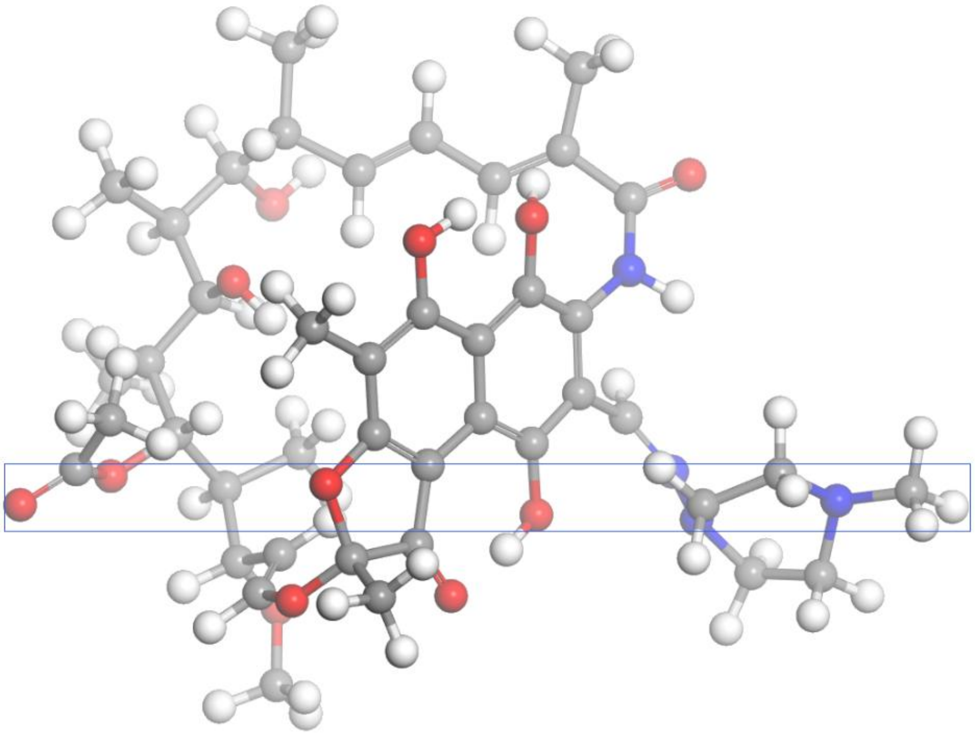

Supplementary Fig. 4. The structure of RFP at the level of 6-311G by using ORCA program. The distance between the two atoms at the edge of the blue wire frame is 17.96 Å. The red ball represents Oxygen atom, ball represents hydrogen atom, Grey represents carbon atoms, Blue represents nitrogen atom. Source data are provided as a Source Data file.

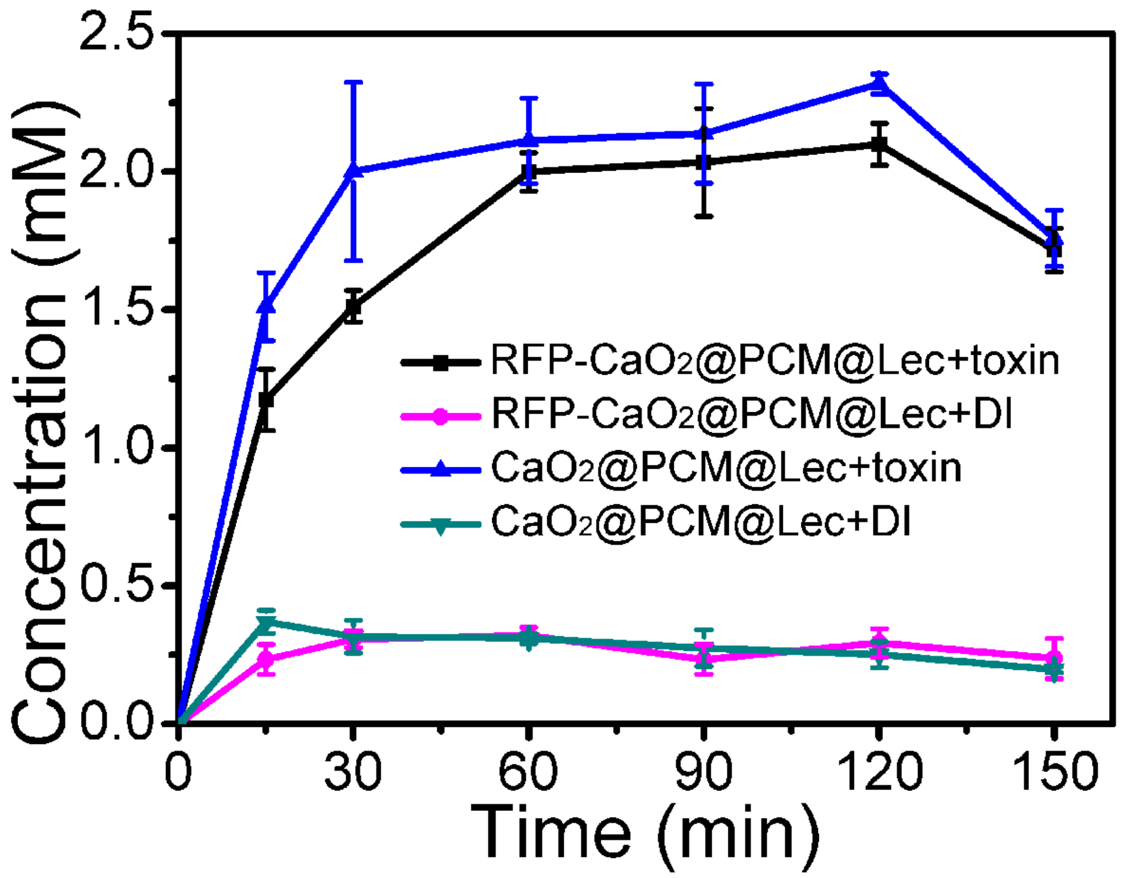

Supplementary Fig. 5. The concentration of H_2O_2 produced by RFP-CaO_2@PCM@Lec (1mg mL^{-1}) and CaO_2@PCM@Lec (1 mg mL^{-1}) at different time (30, 60, 90, 120,150 min) and different treatment conditions (add the toxin or the deionized water). Error bars = standard deviation (n=3). Source data are provided as a Source Data file.

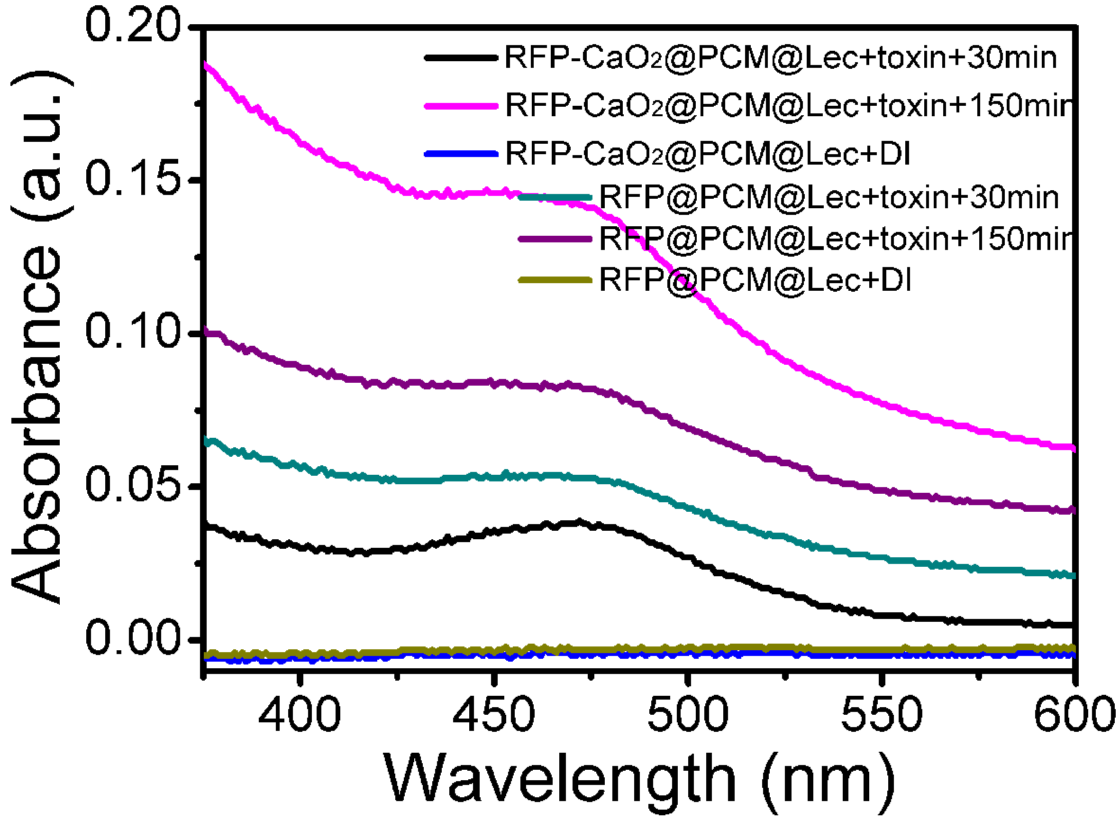

Supplementary Fig. 6. UV absorption spectra of RFP. The RFP release from the RFP-CaO_2@PCM@Lec and RFP@PCM@Lec incubated with toxin and DI at 37 ℃ for different periods of time. (30, 60, 90, 120,150 min). Source data are provided as a Source Data file.

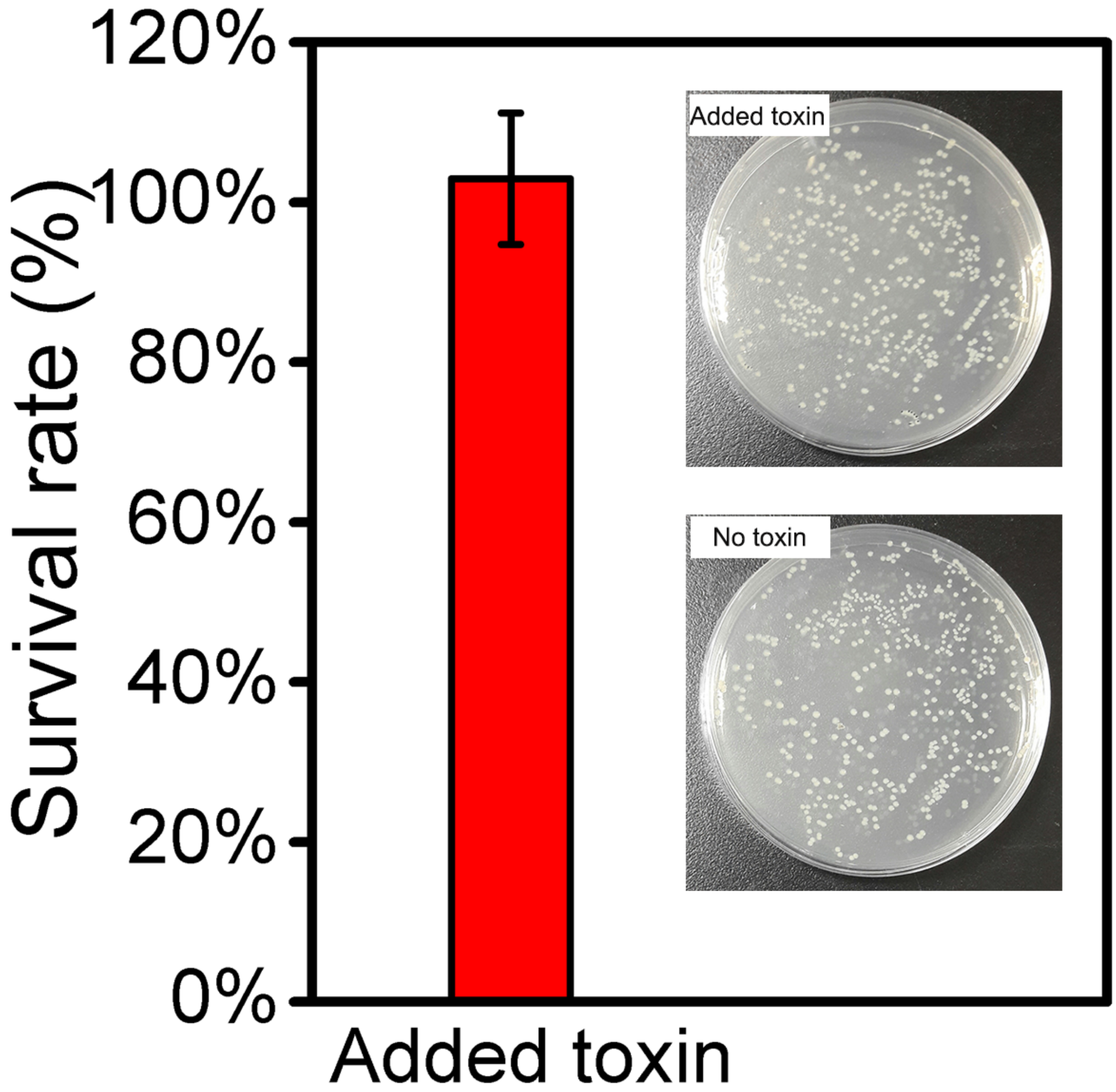

Supplementary Fig. 7. Toxicity of toxins to *B. Subtilis,* the survival rate(%)=$CFU_{(Added\ toxin\ group)}/CFU_{(No\ toxin\ control\ group)}$ ×100%,. Error bars = standard deviation (n=3). Source data are provided as a Source Data file.

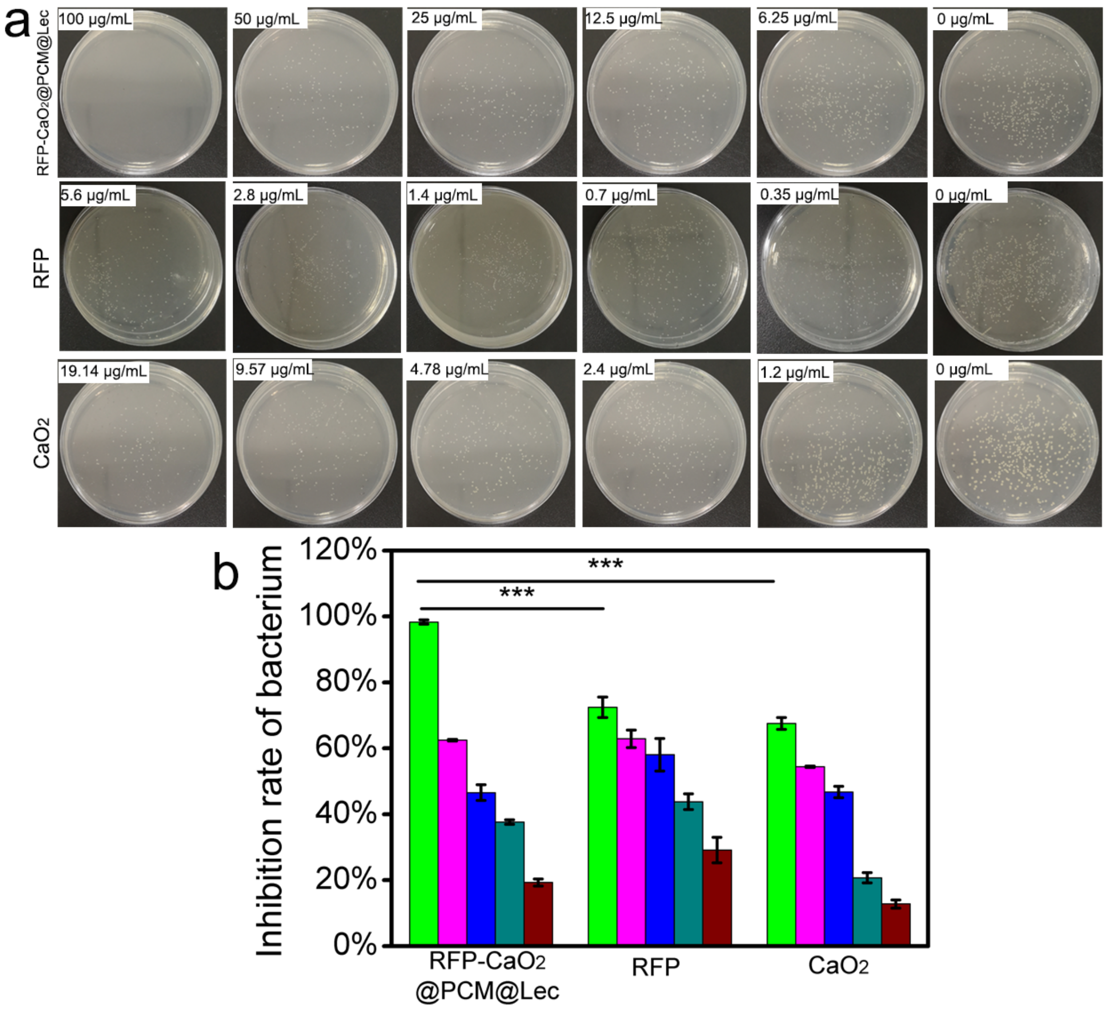

Supplementary Fig. 8. The antibacterial activity of different nanomaterials. Coated flat panel (a) and Bacterial inhibition rate of MRSA incubated with different concentrations of RFP-CaO_2@PCM@Lec (100, 50, 25, 12.5, 6.25, 0 μg mL^{-1}), RFP(5.6, 2.8, 1.4, 0.7, 0.35, 0 μg mL^{-1}) and CaO_2 (19.14, 9.57, 4.78, 2.4, 1.2, 0 μg mL^{-1}). Error bars = standard deviation (n=3). The mean value was calculated by the t test (mean ± SD). *$p < 0.05$, **$p < 0.01$, ***$p < 0.001$, compared with the indicated group. Source data are provided as a Source Data file.

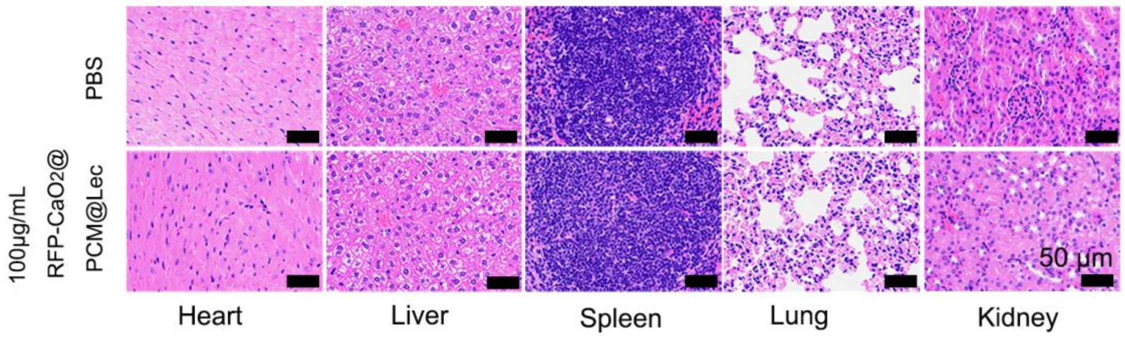

Supplementary Fig. 9. Histological analysis of internal organs (heart, liver, spleen, lung, kidney) injury in the given dose nanoreactors. Source data are provided as a Source Data file.

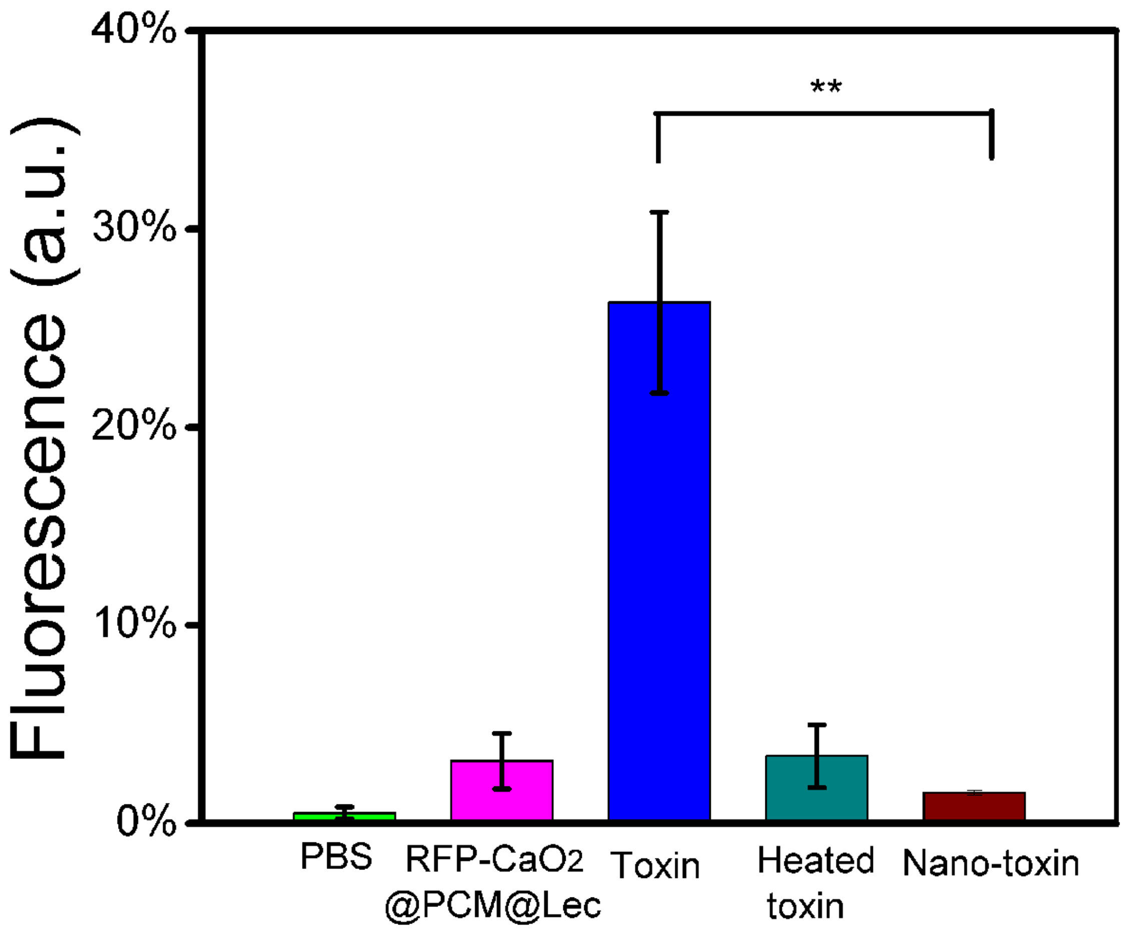

Supplementary Fig. 10. The quantitative analysis of TUNEL staining. Error bars = standard deviation (n=3). The mean value was calculated by the t test (mean ± SD). $*p < 0.05$, $**p < 0.01$, $***p < 0.001$, compared with the indicated group. Source data are provided as a Source Data file.

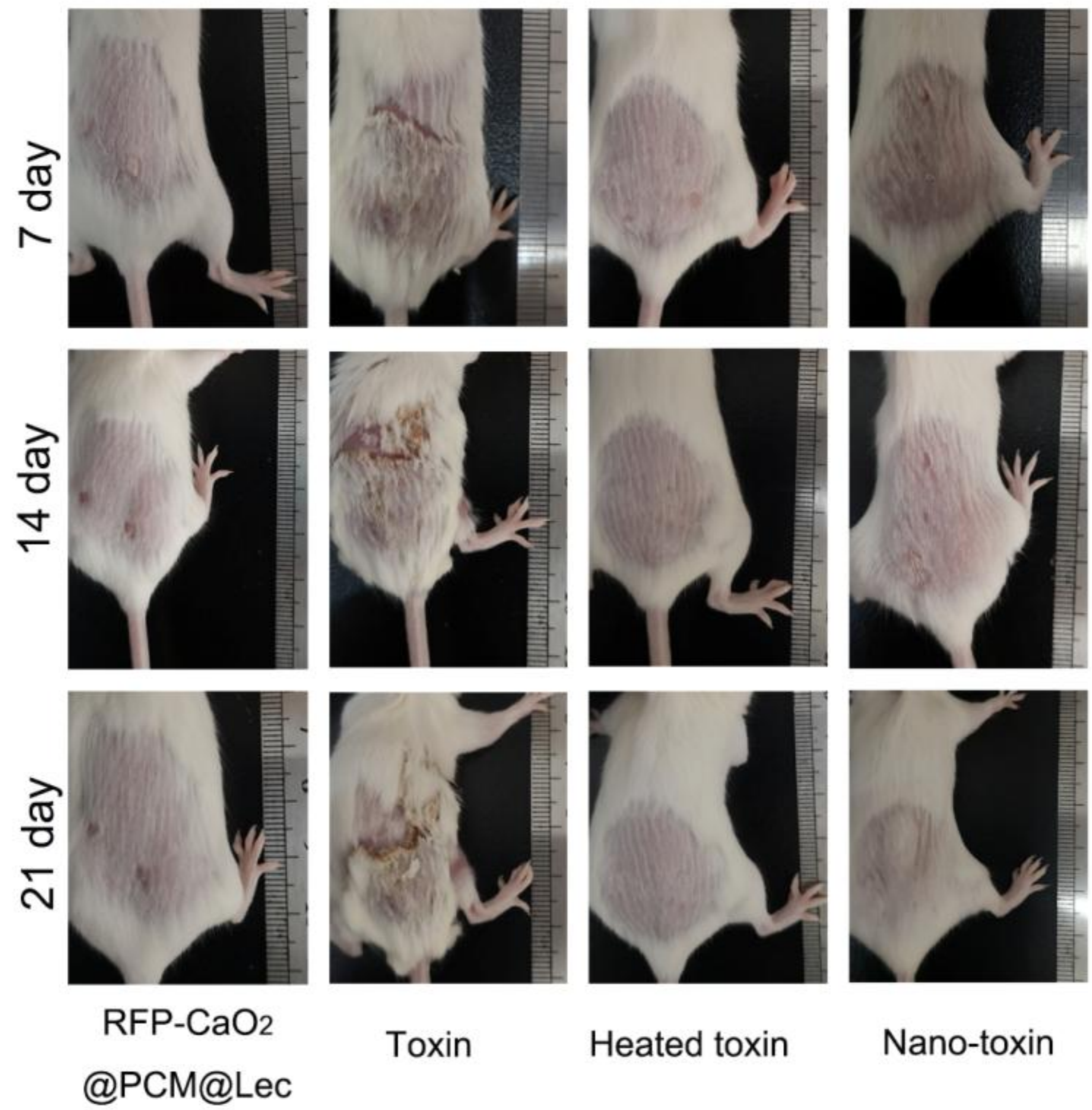

Supplementary Fig. 11. In vivo toxin neutralization. Mice injected with RFP-CaO_2@PCM@Lec, heated toxin, toxin and nano-toxin. Skin lesions occurred 7, 14, 21days following injection. Source data are provided as a Source Data file.

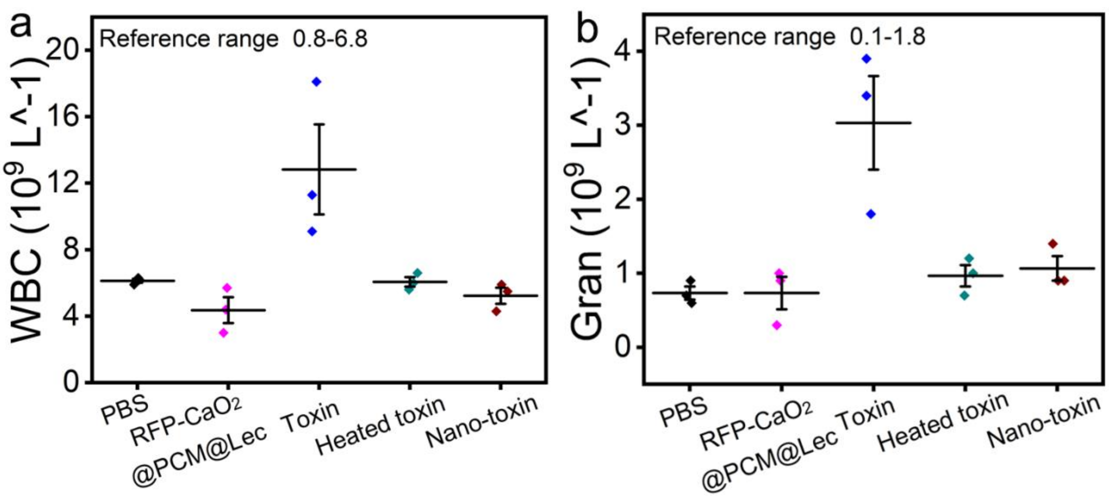

Supplementary Fig. 12. Mice injected with RFP-CaO_2@PCM@Lec, heated toxin, toxin and nano-toxin. After 21 days, the blood routine changes in mice. (a) white blood cells (WBC) and (b) neutrophils (Gran). Error bars = standard deviation (n=3). Source data are provided as a Source Data file.

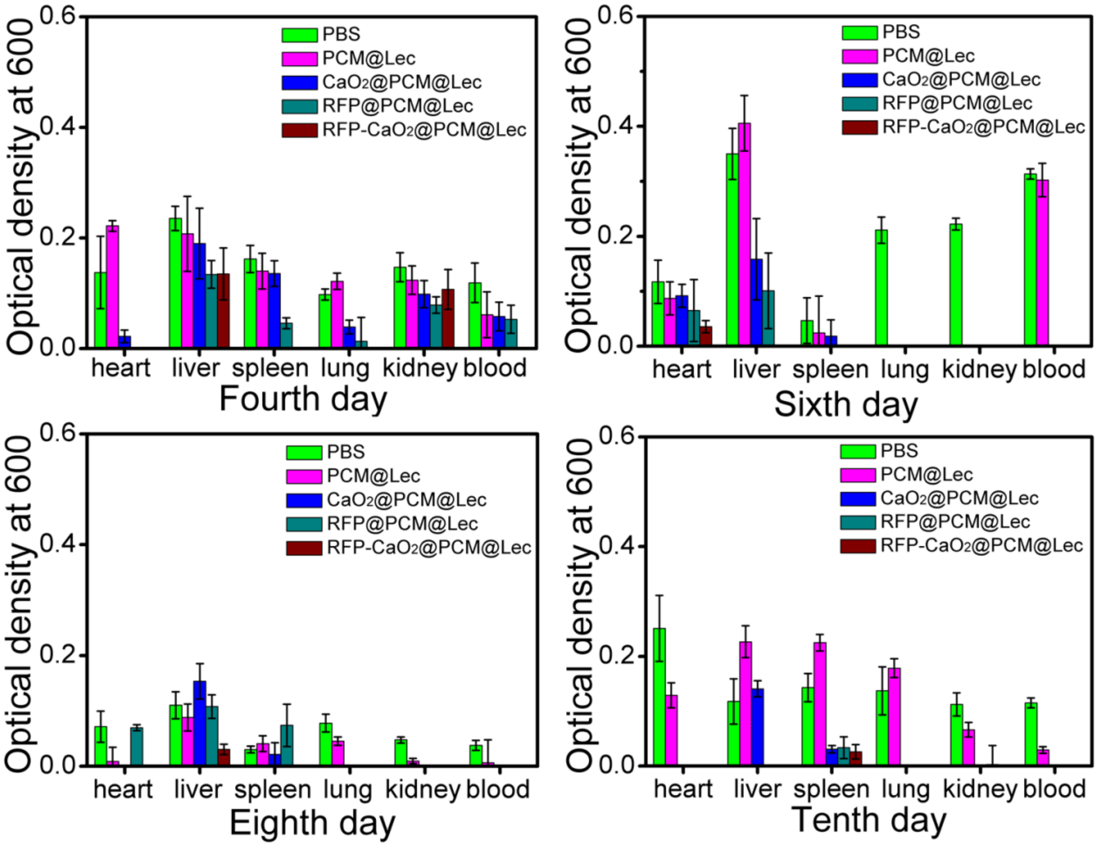

Supplementary Fig. 13. The number of bacteria in the organs (heart, liver, spleen, lung, kidney) and blood of mice by turbidity method for different days. Error bars = standard deviation (n=3). Source data are provided as a Source Data file.

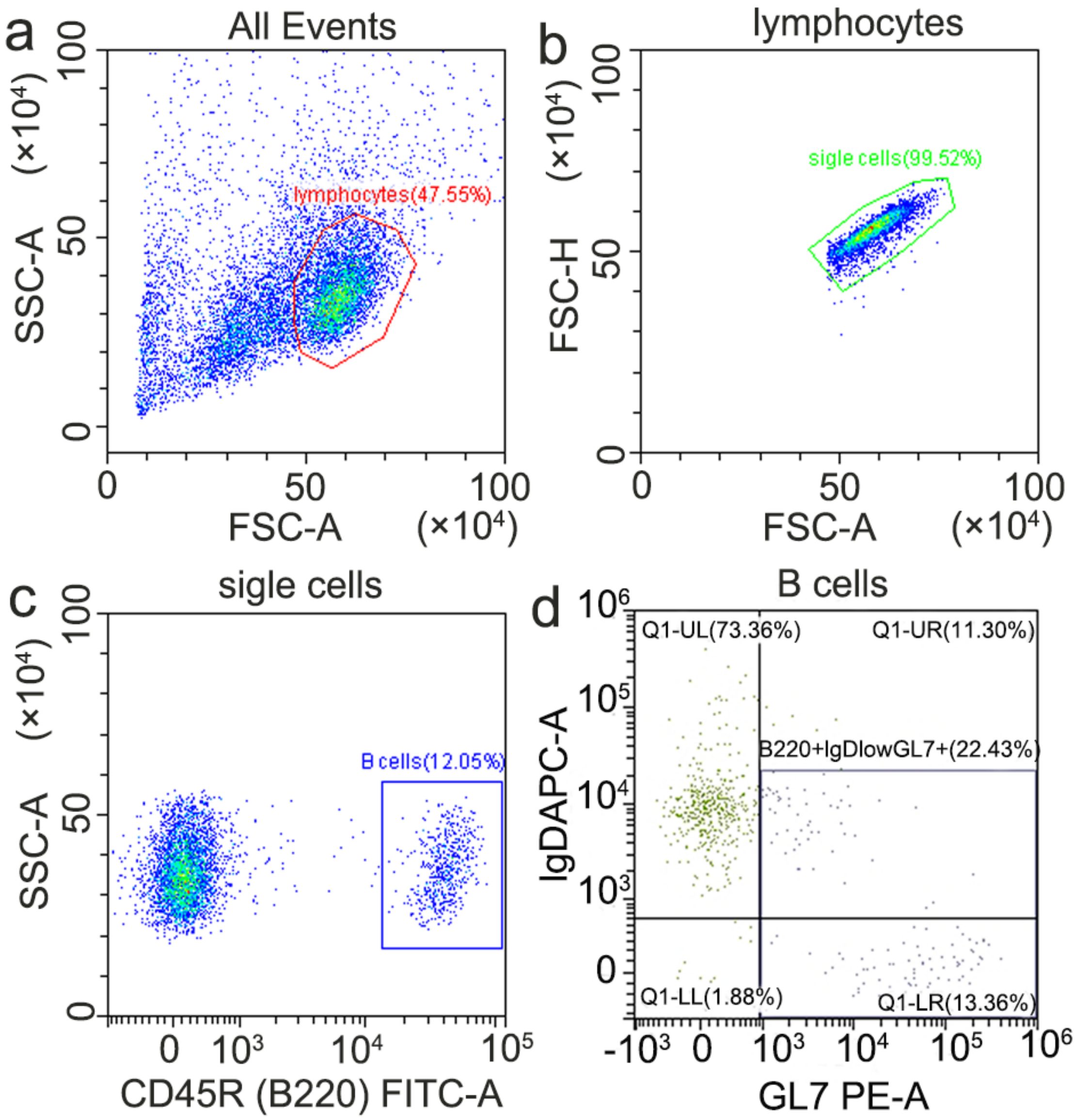

Supplementary Fig. 14 Gating strategy for detection of GL-7 B cell.

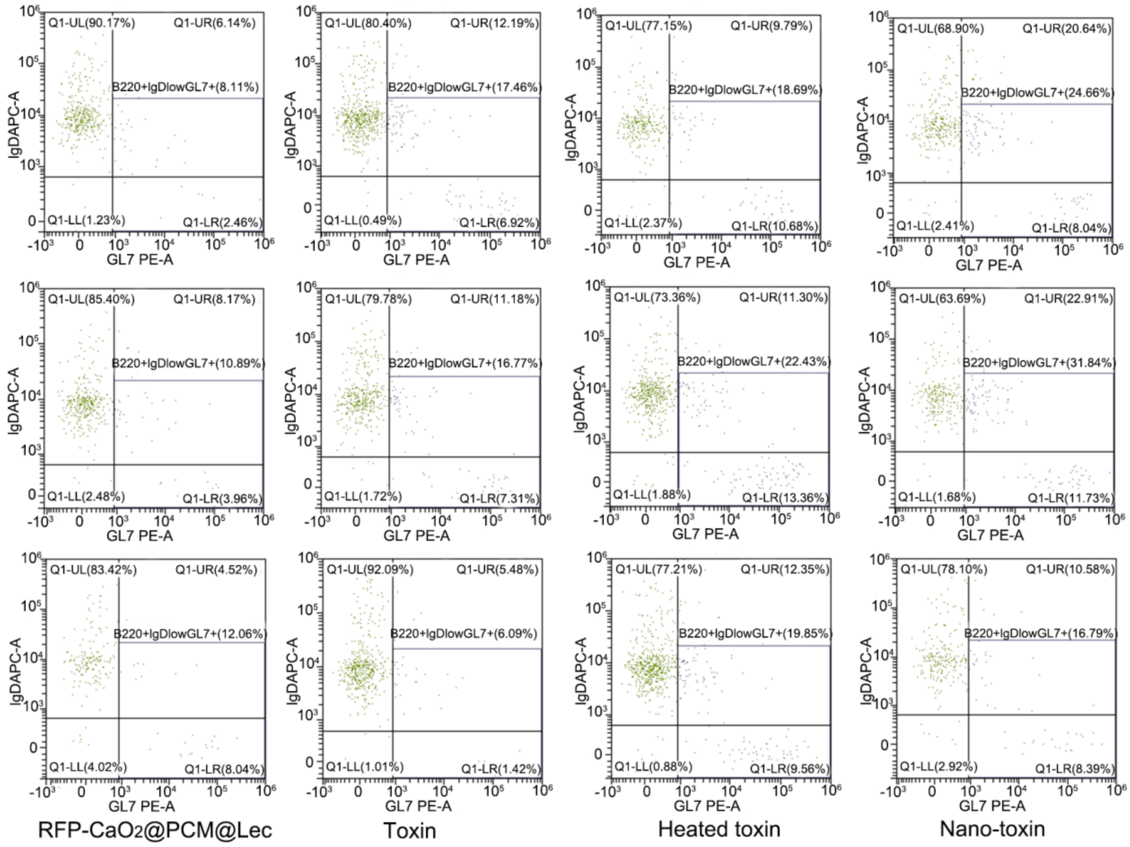

Supplementary Fig. 15. The flow cytometric data of different treatment. Source data are provided as a Source Data file.

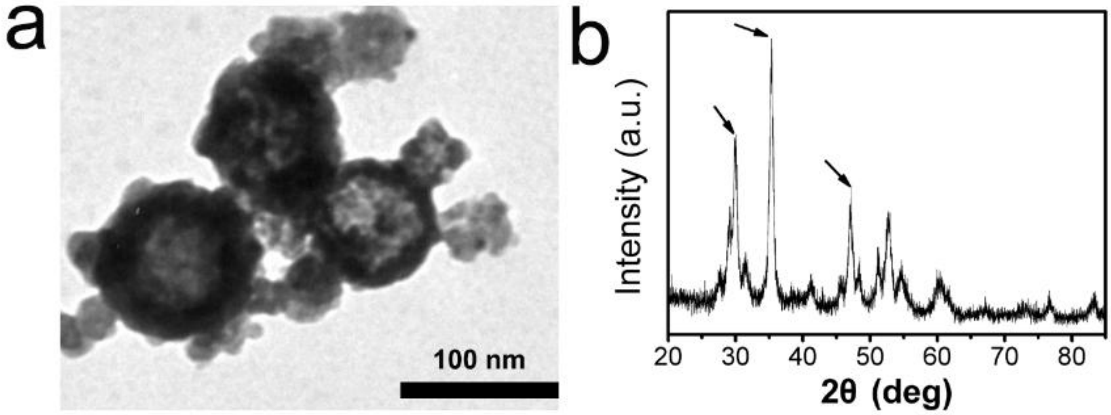

Supplementary Fig. 16. Characterization of CaO_2. (a) TEM and (b) XRD. Source data are provided as a Source Data file.

Supplementary Table 1. The melting temperature of different ratio LA to SA. Source data are provided as a Source Data file.

Mass ratio (LA:SA)		Melting temperature range (℃)
0.0	100.0	71.8-72.3
10.0	90.0.	69.2-71.7
20.0	80.0	65.8-69.7
30.0	70.0	61.3-66.7
40.0	60.0	64.1-69.0
50.0	50.0	61.4-67.3
60.0	40.0	59.4-65.6
65.0	35.0	37.6-38.1
70.0	30.0	36.5-38.8
77.5	22.5	35.1-39.4
80.0	20.0	35.2-38.3
82.5	17.5	37.1-43.8
85.0	15.0	35.7-40.0
90.0	10.0	34.9-40.0
100.0	0.0	45.7-46.2

Supplementary Table 2. The loading rate of RFP and CaO_2 for different materials.

Source data are provided as a Source Data file.

Materials	Loading rate of RFP (%)		Loading rate of CaO_2(%)	
	Before filtering	After filtering	Before filtering	After filtering
RFP-CaO_2@PCM@Lec	8.5%±0.1%	5.4%±0.9% (*)	20.6%±3.3%	17.2%±1.2%
RFP@PCM@Lec	10.9%±4.0%	8.2%±1.1%	0	0
CaO_2@PCM@Lec	0	0	28.2%±3.1%	21.9%±1.8%(*)
PCM@Lec	0	0	0	0

Supplementary References

1. Zhou, X. F., Zhang, Y. L., Qian, Y. J., Zhang, J., Chen, J. B., Gao, H. P. Preparation of nano-calcium peroxide by Chemical Modification. CN-A 103601155, (2014).
2. Khodaveisi, J., Banejad, H., Afkhami, A., Olyaie, E., Lashgari, S., Dashti, R. Synthesis of calcium peroxide nanoparticles as an innovative reagent for in situ chemical oxidation. *J. Hazard. Mater.* 192, 1437-1440 (2011).
3. Wei, X. et al. In situ capture of bacterial toxins for antivirulence vaccination. *Adv. Mater.* 29, 1701644-1701651 (2017).
